# Prevalence and factors associated with teenage pregnancy among parturients in Mbale Regional Referral Hospital: a cross sectional study

**DOI:** 10.4314/ahs.v22i2.52

**Published:** 2022-06

**Authors:** Violet Chemutai, Milton W Musaba, Dinah Amongin, Julius N Wandabwa

**Affiliations:** 1 Department of Public and Community Health, Busitema University, Mbale, Uganda; 2 Department of Obstetrics and Gynecology, Busitema University, Faculty of Health Sciences Mbale, Uganda; 3 Department of Health Policy Planning and Management, Makerere University School of Public Health

**Keywords:** Teenage pregnancy, associated factors, Eastern Uganda

## Abstract

**Introduction:**

In Uganda, 12% of the audited maternal deaths occur among teenagers. The prevalence and factors associated with teenage pregnancy in health facilities is not well documented. We determined the prevalence and factors associated with teenage pregnancy in Mbale Regional Referral Hospital.

**Materials and Methods:**

We conducted a cross sectional study between July and September 2019.We consecutively enrolled and collected information from 427 respondents. Associated factors were determined using logistic regression, a P-value < 0.05.

**Results:**

The prevalence of teenage pregnancy was 20.6% [95% CI : (17.0% – 24.7%)]. Rural residence [(AOR: 2.8 (1.08 – 7.09)], primary level of education [(AOR: 9.57 (3.48 – 26.3)], unhappy feelings about the current pregnancy [(AOR: 3.57(1.05 – 12.15)], primiparity [(AOR: 21.05 (7.36 – 60.15)] increased the likelihood of teenage pregnancy. While, history of ever use of contraceptives [(AOR: 0.32 (0.12 – 0.84)], age at coitarche [(AOR: 0.68 (0.54 – 0.85)], grand multiparity [(AOR: 0.06 (0.01 – 0.51)], and exposure to sex education at home [(AOR: 0.37 (0.15 – 0.89)] decreased its likelihood.

**Conclusion:**

The prevalence of teenage pregnancy was high. It was associated with being resident in rural area, low level of education and ever use of contraception. Promotion of reproductive health education may prevent teenage pregnancy.

## Introduction

Teenage pregnancy is defined as any pregnancy occurring between the age of 10–19 years 1,2. It's estimated that 21 million girls aged 15 to 19 years and 2 million girls less than 15 years become pregnant every year 2,3. About 16 million of these girls give birth each year, mostly (90%) in low income countries 4. At 25%, Uganda has one of the highest teenage pregnancy rates in sub-Saharan Africa^5^. The prevalence of teenage pregnancy is very variable, it ranges from 2.3 to 19.2% in south Africa, 31% in Kenya, 20.4 in Ethiopia and 31% in Sudan 6.

Various sociodemographic, cultural, and individual factors have been associated with teenage pregnancy 6. The factors include, early marriage, poverty, type of occupation, religious beliefs, peer pressure and place of residence^7^. In this regard, the government of Uganda has undertaken several multisector interventions to prevent teenage pregnancies. Programs such as the universal primary and secondary education aim at empowering and keeping the girl child in school for a longer period of time to prevent early marriages; enactment of laws against defilement and childhood marriages are intended to deter the actors and perpetrators of this vice 8. Despite all these efforts by government, 12% of the audited maternal deaths occur among teenage mothers 9.

Compared to women in their twenties, teenage girls are twice more likely to die in pregnancy and childbirth^3^,^10^,^11^, this risk increases five fold for those bellow the age of 15 years^12^. Teenage mothers are more likely to suffer adverse pregnancy outcomes including death because they are not biologically and psychologically ready for childbirth, and they have limited or no access to quality maternity care because of social and economic disadvantages 13. These complications include obstructed labor and its sequelae, pregnancy related hypertension, anemia, low birth weight, fetal growth retardation, and psychological trauma all leading to high infant and maternal morbidity and mortality rate 2,4. Teenage pregnancy and child bearing therefore has far reaching short- and long-term negative effects for the teen parents, their offspring's and the community at large 14.

Given the substantial variations in health care seeking patterns in this clinically important age group 15,16, there is need for accurate epidemiological information on teenage pregnancy at the health facility level. In Uganda, previous estimates of prevalence and associated factors have mostly used secondary data from demographic health surveys as well as community studies. Consequently, the burden of teenage pregnancy in health facilities is not well documented. Therefore, this study aimed to determine the prevalence and factors associated with teenage pregnancy among women admitted in active labour at Mbale Regional Referral Hospital.

## Materials and methods

### Study setting

The study was conducted in Mbale Regional Referral Hospital in Eastern Uganda. It is a 400-bed capacity hospital serves about 14 districts in the Elgon zone and its surroundings with an estimated population of 2,500,000 people per year. The districts include Bukwo, Bukedea, Budaka, Kibuku, Kumi, Kween, Kapchorwa, Sironko, Manafwa, Tororo, Namisindwa, Bududa, Parts of Nakapiripirit and Mbale. Mbale hospital offers specialized healthcare services and also serves as a teaching hospital for medical interns, medical students from the nearby training institutions. The department of obstetrics and gynecological is divided into units; labor ward has six delivery beds, high dependency unit with four beds, postnatal ward has 30 beds, maternity one which is a gynecological ward has 23 beds and a minor theatre with one operation bed. Others include Antenatal clinic, family planning, and gynecological outpatient clinics. There is no stand alone clinic for adolescents in the hospital; they are served together with the adults. About 14% of the 8,000 mothers that give birth in the labour ward annually are below the age of 20 years.

### Study design

This was a cross sectional study conducted between July and September 2019

### Study population

All parturients admitted to Mbale Regional Referral Hospital for childbirth were the source population of the study.

### Sample size and sampling

We used the formula for determining sample size for frequency in a population as described in Open Epi, Version 3, open source calculator^17^. We assumed that the percentage frequency of the outcome factor (proportion of teenage pregnancies) in Mbale Regional Hospital (p) was 50% since we could not find any similar studies conducted in a health facility setting. We further assumed a design effect (DEFF) of one (1), a power of 95% and 95% confidence level. After correcting for a non-response rate of 10%, it was estimated that a sample size of 427 would be sufficient to determine the associated factors. Over a period of three months, we consecutively enrolled parturient women admitted to labour ward on a daily basis until the estimated sample size was achieved.

### Inclusion criteria

We included all parturient women who were admitted and delivered in the last 24 hours before enrollment.

### Exclusion criteria

We excluded all those parturient women that were critically ill and therefore not in position to give a written informed consent.

### Study variables

Our outcome variable was the proportion of parturient women below the age of 20 years. For the exposure variables, we collected information on a variety of factors from the point of recruitment up to the time of discharge from the hospital. The sexual and reproductive health factors included exposure to sexual education information at home categorized as yes or no, age of menarche and coitarche in years, history of ever use of contraception, whether the pregnancy was planned or unplanned and feelings about the current pregnancy). The socio economic and cultural factors were maternal age in years, marital status categorized as married or not married, place of residence categorized as rural or urban, level of education categorized as primary or post primary as well as occupation of respondent.

### Data collection

Between July and September 2019, we consecutively enrolled all eligible parturient women that consented to participate in this study until the estimated sample size was attained. The principal investigator and his team of three well-trained midwives obtained a written informed consent from each of the respondents before enrollment. An interviewer-administered questionnaire was used to collect data by a team of three well-trained research assistants, who were also qualified midwives employed in Mbale Regional Referral Hospital. The questionnaire was administered at different time points from enrollment to discharge from the hospital. Research assistants that spoke the same local language, mainly Lumasaba, Luganda, Lugwere and Ateso, administered questions in the participants' local dialect. The tool was translated into different languages before data collection commenced.

### Data management

In order to minimize errors at entry, two different people familiar with the software entered the data into a computer using Epi Data software Version 3.1. It was then exported to STATA Version 14.2 for cleaning and analysis. Before analysis data were coded, value labels were defined, edited and data manipulations were done. Data security was ensured by having passwords on the personal computers to ensure that no person accessed the data without permission.

### Data analysis

The prevalence of teenage pregnancy was calculated as a proportion of mothers below the age of 20 years and expressed as percentage of the entire study sample population. Categorical variables were summarized as frequencies with their percentages while the continuous variables were summarized as means with their standard deviations (SD). The primary outcome was cross tabulated with various independent variables at bivariable analysis to determine associations using the Chi squared test. All variables with a P-Value of at least 0.25^18^ from bivariable analysis or known to be risk factors from literature were included in the final multivariable model in a stepwise logistic regression model. Adjusted odds ratios with a 95% confidence interval were computed, and variables with a p-value <0.05 in the final Model were considered as statistically significant.

## Results

### Baseline characteristics of the study participants

The mean age of all the respondents was 25.4 (6.2) years. Among the teenagers, the mean age was 17.7 years (SD=1.2) and 27.3 (5.3) years among the non-teenagers. Majority of the respondents were married (367/427) 86% and resident in the rural areas 261/427. About (222/427) 60% of participants had attained post primary education, while (235/427) 55% of participants reported non-contraceptive use. The details are in table 1.

**Table 1:** Characteristics of the study participants

	Total (%)	Teenage pregnancy (%)	
Characteristic	(N=427)	yes (n=88)	no (n=339)
**Mean age, years (SD)**	25.4 (6.2)	17.7 (1.2)	27.3 (5.3)
**Mean age of menarche, years (SD)**	14.7 (1.8)	14.3 (1.3)	14.8 (1.8)
**Mean age at coitarche, years (SD)**	16.8 (2.5)	15.5 (1.4)	17.1 (2.6)
**Marital status**			
Not married	60 (14)	35 (40)	25 (7.4)
Married	367 (86)	53 (60)	314 (92.6)
**Place of residence**			
Rural	261 (61.1)	70(80)	191 (56.3)
Urban	166 (38.9)	18 (20)	148 (43.7)
**Education level**			
Primary	205 (48)	66(75)	139 (41)
Post primary	222 (60)	22 (25)	200 (59)
**Occupation**			
Peasant farmer	85 (20)	21 (24)	64 (19)
House wife	152 (36)	119 (35)	33 (38)
Retail business	83 (19)	5 (6)	78 (23)
Student	42 (10)	19 (22)	23 (7)
Paid employee	65 (15)	10 (11.4)	55 (16.2)
**Use of contraceptives**			
Yes	192 (45)	71 (81)	121 (36)
No	235 (55)	17 (19)	218 (64)
**Pregnancy planned**			
Yes	160 (37.5)	56 (64)	104 (31)
No	267 (62.5)	32 (36.5)	235 (69)
**Feeling about pregnancy**			
Not happy	81 (19)	49 (56)	42 (12.4)
Happy	346 (81)	39 (44)	297 (87.6)
**Parity**			
Para 1	133 (32)	72 (84)	61 (18.2)
Para 2 to 4	198 (47)	13 (15)	185 (55.1)
Para 5+	91 (22)	1(1)	90 (26.8)
**Alcohol consumption**			
yes	192 (45)	20 (22.7)	109 (32.2)
no	235 (55)	68 (77.3)	230 (67.9)
**Sex education at home**			
Yes	280 (66)	63 (72)	217 (64)
No	147 (34)	25 (28)	122 (36)
**Parents education level**			
Primary	209 (49)	47 (53.4)	162 (48)
Post primary	218 (51)	41 (46.6)	177 (52)

### Prevalence of teenage pregnancies

The prevalence of teenage pregnancy among parturients in Mbale Regional Referral Hospital was (88/427) 20.6% [95% CI: (17.0% - 24.7%)]. The details are in table 1.

### Factors associated with teenage pregnancy

In the adjusted analysis, the factors associated with teenage pregnancies included: age of coitarche, for each additional year that a girl delayed sexual debut, the likelihood of having a teenage pregnancy reduced by 0.7 times [(AOR=0.68, (95% CI: 0.54, 0.85)]. The teenage mothers were 10 times more likely to have stopped at the primary level of education compared to those that were 20 or more years of age. [(AOR=9.57, (95%CI 3.48, 26.30)]. Teenage mothers were 0.3 times less likely to have ever used contraception (AOR= 0.32, 95% CI (0.12, 0.84) and 0.4 times less likely to have been exposed to sex education information at home [(AOR=0.37 (95% CI: 0.15, 0.89 compared to the older parturients. The rest of the details are in table 2.

**Table 2:** Factors associated with teenage pregnancy in Mbale district Eastern Uganda

	Teenage pregnancy	Crude		Adjusted	
		
Characteristic	Yes/No	odds Ratio (95% CI)	P-Value	odds Ratio (95% CI)	P-Value
**Mean age of menarche,** **years (SD)**	14.3/14.8	0.83 (0.71 -0.96)	0.012	0.86 (0.65 -1.14)	0.307
**Mean age at coitarche,** **years (SD)**	15.5/17.1	0.70 (0.61 -0.79)	< 0.001	0.68 (0.54 -0.85)	**0.001**
**Marital status**					
Not married	35 /25	1		1	
Married	53 /314	0.12 (0.67 -0.22)	< 0.001	0.80 (0.24 -2.70)	0.725
**Place of residence**					
Rural	70/191	3.01 (1.72 -5.28)	< 0.001	2.8 (1.08 -7.09)	**0.033**
Urban	18 /148	1		1	
**Education level**					
Primary	66/139	4.32 (2.54 -7.32)	< 0.001	9.57 (3.48 -26.30)	**< 0.001**
Post primary	22 /200	1		1	
**Occupation**					
Peasant farmer	21 /64	1.18 (0.63 -2.21)	0.598	0.49 (0.17 -1.43)	0.189
House wife	119 /33	1		1	
Retail business	5 ,78	0.23 (0.09 -0.62)	0.003	0.39 (0.09 -1.66)	0.204
Paid employee	5,55	0.66 (0.30 -1.43)	0.287	0.64 (0.15 -2.76)	0.545
student	19/23	2.10 (1.45 -6.11)	0.003	1.66 (0.41 -6.69)	0.477
**Use of contraceptives**					
Yes	71 /121	0.13 (0.07 -0.24)	< 0.001	0.32 (0.12 -0.84)	**0.021**
No	17 /218	1		1	
**Pregnancy planned**					
Yes	56 /104	0.25 (0.15 -0.41)	< 0.001	0.80 (0.31 -2.07)	0.645
No	32 /235	1		1	
**Feeling about pregnancy**					
Unhappy	49 /42	1		1	
Happy	39 /297	5.63 (3.31 -9.57)	< 0.001	3.57 (1.05–12.15)	**0.042**
**Parity**					
Para 1	72 /61	16.80 (8.70 -32.42)	< 0.001	21.05(7.36–60.150	**< 0.001**
Para 2 to 4	13 /185	1		1	
Para 5+	1/90	0.16 (0.20 -1.23)	0.078	0.06 (0.01 -0.51)	**0.01**
**Alcohol consumption**					
Yes	20 ,109	1.61 (0.93 -2.79)	0.088	1.68 (0.69 -4.14)	0.256
no	68 /230	1		1	
**Sex education at home**					
Yes	63 /217	0.71 (0.42 -1.18)	0.184	0.37 (0.15 -0.89)	**0.026**
No	25 /122	1			
**Parents education level**					
Primary	47 /162	1.25 (0.78 -2.00)	0.348	_	_
Post primary	41 /177				

## Discussion

We investigated the prevalence and factors associated with teenage pregnancy among parturients admitted to the labour ward in Mbale Regional Referral Hospital, using a cross-sectional study design. We found a high prevalence of teenage pregnancy and it was associated with rural residence, level of education, unhappy feelings about the pregnancy, parity, history of ever use of contraceptives, age at coitarche and exposure to sex education information at home.

The prevalence of teenage pregnancy among parturients in this study was lower than what was reported at population level in Eastern Uganda. Using data from the Uganda Demographic and Health Survey of 2011, the prevalence of teenage pregnancy was found to be 35% in Eastern Uganda compared to our finding of 21%. This current study was undertaken at one referral facility, which may partly explain the observed reduction in rates of teenage pregnancy. This is in addition to the fact that there has been an improvement in the literacy levels because of improved access to education through the free universal primary and secondary education programs in the country^5^,^19^. Improved literacy is a factor known to promote utilisation of sexual reproductive health services^5^,^19^. Nonetheless, this prevalence is still high and consistent with findings from a systematic review of adolescent pregnancies in Africa, which revealed that East Africa had the highest rate at 21.5% 20. Some of the reasons for the high prevalence of teenage pregnancy include inaccessibility of contraceptive services, the unfavorable attitude of the community towards the adolescent, contraceptives, poor knowledge of adolescents of the SRH) issues and widespread sexual violence against girls that is still perceived as normal occurrence in the predominantly patriarchal societies^20^.

Second, we found that participants that delayed their first sexual contact, had exposure to sex education information at home and ever used contraception were less likely to be teenage mothers. This finding aligns with previous research and survey findings that improved access to quality adolescent friendly sexual and reproductive health services is an effective low cost intervention against teenage pregancies 21,22. This calls for the need to advocate for adolescent sexual reproductive health rights by promoting access to information both at home and school.

Third, we found that those who had attained a maximum of primary level of education and had unhappy feelings about the current pregnancy were more likely to be teenagers. These results affirm previous research findings that young girls who leave school early are more likely to end up with a teenage pregnancy 23,24. Girls that acquire education are most likely to use effective contraceptives than their less achieving counterparts and are generally empowered to make wiser choices regarding their health 25. Contraception needs and use among teenagers regardless of their marital status is still a major concern. This was also noted in Malaysia where information about contraception is not widely discussed with teenagers^26^, leading to limited access to contraception and more unintended pregnancies. Teenage girls need a lot of support in terms of sexual education in order to prevent unplanned pregnancies. Therefore, there is need for sustained campaigns to promote free access to sexual reproductive education in this age group. The universal primary and secondary education program in Uganda should be supported and continually improved because it is vital intervention strategy.

Lastly, teenage mothers were likely to be resident in rural areas with no history of ever use of contraception. This is consistent with findings from a systematic review on adolescent pregnancy in Africa 20, which found rural residence to be a strongly associated with teenage pregnancy. In contrast, a review of the literature on determinants of first time pregnancy found that adolescents who lived in rural areas had lower odds of first adolescent pregnancy^25^. The authors postulated that individual level factors play a role in the association between place of residence and first adolescent pregnancy. This suggests the role of limited or no access to social services like health care and education in the provision of sexual and reproductive services. Limited exposure to mass media (television, radio) and social media has also been shown to negatively affect access to sexual and reproductive health information by girls that reside in rural areas 25.

## Methodological considerations

This study was adequately powered to determine the prevalence and factors associated with teenage pregnancy among parturients in busy regional referral hospital. This was a facility-based study undertaken in a referral hospital where most of the high-risk pregnancies including adolescents are referred, so our estimate may be higher. Additionally we could not establish causality and independent risk factors because of the nature of the study design, these are just associations.

## Conclusion

In Mbale hospital, there is a high prevalence of teenage pregnancy. The study identified rural residence, low level of education as predictors of teenage pregnancy and ever used contraceptive as protective for teenage pregnancy. Education of the girl child, promotion of sexual education and availability of contraceptives in health units may lead to reduction of teenage pregnancy rates. This baseline information is essential for the design of future interventions to reduce the burden of teenage pregancies and the related squale.

## Data Availability

Data supporting the results reported in this manuscript can be obtained from this link http://dx.doi.org/10.17632/4tpkb43wzy.1.
